# S-Fertilizer (Elemental Sulfur) Improves the Phytoextraction of Cadmium through *Solanum nigrum* L.

**DOI:** 10.3390/ijerph19031655

**Published:** 2022-01-31

**Authors:** Aishah Alatawi, Xiukang Wang, Arosha Maqbool, Muhammad Hamzah Saleem, Kamal Usman, Muhammad Rizwan, Tahira Yasmeen, Muhammad Saleem Arif, Shamaila Noreen, Afzal Hussain, Shafaqat Ali

**Affiliations:** 1 Biology Department, Faculty of Science, University of Tabuk, Tabuk 71421, Saudi Arabia; Amm.alatawi@ut.edu.sa; 2 College of Life Sciences, Yan’an University, Yan’an 716000, China; 3 Department of Environmental Sciences and Engineering, Government College University, Faisalabad 38000, Pakistan; aroshamaqbool@gmail.com (A.M.); mrizwan@gcuf.edu.pk (M.R.); tahirayasmeen@gcuf.edu.pk (T.Y.); msarif@gcuf.edu.pk (M.S.A.); shamailanoureen@gcuf.edu.pk (S.N.); afzal.hussain@envs.uol.edu.pk (A.H.); 4 College of Plant Science and Technology, Huazhong Agricultural University, Wuhan 430070, China; saleemhamza312@webmail.hzau.edu.cn; 5 Agricultural Research Station, Office of VP for Research & Graduate Studies, Qatar University, Doha 2713, Qatar; kusman@qu.edu.qa; 6 Department of Environmental Sciences, The University of Lahore, Lahore 54590, Pakistan; 7 Department of Biological Sciences and Technology, China Medical University, Taichung 40402, Taiwan

**Keywords:** medicinal plant, element, gypsum, heavy metal, phytoremediation

## Abstract

Soil contamination with toxic heavy metals [such as cadmium (Cd)] is becoming a serious global problem due to the rapid development of the social economy. This study was carried out to assess the beneficial role of two different kinds of (S)-fertilizer in the phytoremediation of Cd contaminated soil through *Solanum nigrum* L. Gypsum (Gyp) and Elemental sulfur (ES) was applied alone and in combination with different ratios (0, 100:0, 0:100, 50:50 mg kg^−1^) accompanied by different Cd levels (0, 25, 50 mg kg^−1^). After seventy days of sowing, plants were harvested for determination of growth, physiological characteristics, oxidants and antioxidants, along with Cd uptake from different parts of the plant. Cd toxicity significantly inhibited growth, physiology and plant defence systems, and also increased Cd uptake in the roots and shoots of *Solanum nigrum* L. The application of Gyp 100 mg kg^−1^ boosted plant growth and physiology along with oxidants and antioxidants activity as compared to ES 100 mg kg^−1^ alone, and combine application of *GYP+ES* 50 + 50 mg kg^−1^. The application of ES 100 mg kg^−1^ showed an effective approach to decreasing Cd uptake as compared to Gyp 100 mg kg^−1^. Overall results showed that the combined application of *GYP+ES* 50 + 50 mg kg^−1^ significantly enhanced the phytoremediation potential of *S. nigrum* in Cd contaminated soil. Thus, it is highly recommended to apply the combined application of *GYP+ES* for phytoremediation of Cd contaminated soil.

## 1. Introduction

In recent decades, rapid increases in urbanization and industrialization have caused the excessive release of heavy metals in farmlands, with damaging effects on ecosystems [1,2,3]. Heavy metal accumulation in soils is of great concern in agricultural production due to its adverse effects on food safety and marketability, crop growth due to phytotoxicity, and the environmental health of soil organisms [4,5,6]. On the other hand, there is a shortage of good quality water being used for irrigation, a problem which only becomes worse over time around the world [7,8]. In addition, heavy metals are significant environmental pollutants, and their toxicity is a problem of increasing significance for ecological, evolutionary, nutritional and environmental reasons [9,10]. Some heavy metals are poisonous to plants even at a very low concentration, while other heavy metals may accumulate in plant tissues to moderately abnormal states with no obvious side effects or decrease in yield [11,12]. The growing of the plants in these heavy metal-contaminated areas, causes changes in their metabolism, physiological and biochemical means, which results in metal accumulation, lower biomass generation and reduction in biomass growth [13,14]. Contamination of agricultural soils with cadmium (Cd) has become one of the most toxic and widespread environmental problems [15,16]. In plants, excess Cd typically causes direct or indirect inhibition of various physiological processes, such as respiration, transpiration, photosynthesis, oxidative stress, cell elongation, nitrogen metabolism and uptake of mineral nutrition, finally resulting in growth retardation, leaf chlorosis and reduced biomass [17,18]. In the case of Cd stress, the plant has involved several strategies that can resort to a number of defense systems, such as: (1) immobilization; (2) exclusion; (3) synthesis of phytochelatins; (4) compartmentalization; (5) synthesis of metallothioneins; (6) synthesis of stress proteins; (7) production of stress ethylene [19,20]. Due to its persistent nature, Cd is ranked as the seventh most toxic heavy metal out of 20 metals, and acceptable concentrations in crops range from 0.013 to 0.22 mg kg^−1^ for cereal crops, 0.07–0.27 mg kg^−1^ for fodder and 0.08–0.28 mg kg^−1^ for leguminous plants [12,21]. Moreover, Cd influx in plant cells occurs via ion specific channels and other proteins that mediate transport of ions at the plasmalemma [20,22]. In addition, Cd is indirectly involved in the biological redox reaction, the oxidative burst is produced by increasing the activity of NADPH oxidases, which results in production of extracellular super oxide, peroxide, and intracellular lipid peroxidation [23,24]. Higher metal concentration in plants causes ultra–structural alterations [25,26,27], oxidative stress in plants and increased electrolyte leakage (EL) and malondialdehyde (MDA) concentrations, in addition to induced alterations in antioxidant enzyme activities such as superoxide dismutase (SOD), peroxidase (POD) and catalase (CAT) and ascorbate peroxidase (APX) [28,29,30]. The uptake of mineral nutrients is changed by Cd exposure, which in turn may disturb biochemical structures. However, some nutrients (Ca, Fe, Zn, Si, S etc.) have been found to counteract the toxic effects of Cd stress in plants through the production of phytochelatins [31,32]. Hence, it is urgently required to safeguard plants from Cd toxicity in order to counter the phytotoxicity and oxidative stress triggered by the uptake of Cd in plants.

For this purpose, there are several techniques for removing heavy metals from metal-contaminated soil, such as soil washing, thermal desorption, incineration, stabilization, and soil flushing [33,34]. However, these techniques have many disadvantages, such as cost, they require 24 h monitoring, and they are not an efficient method to remove toxic heavy metals and other contaminants from agricultural land [35]. Phytoremediation is the direct use of living green plants and is an effective, cheap, non-invasive, and environmentally friendly technique used to transfer or stabilize all the toxic metals and environmental pollutants in polluted soil or ground water [7,36]. Furthermore, phytoremediation is concerned with the potential of a plant species to accumulate high concentrations of toxic pollutants in their tissues. Among other macro-nutrients, sulfur (S) improves plant growth and physiology during biotic and abiotic stress [37]. It ranks fourth in the list of vital nutrients after nitrogen, phosphorus, and potassium, and is essential to plant development [38,39]. Sulfur has amazing potential to detoxify the Cd toxicity as compared to other nutrients, and its protective mechanism is well documented [40]. Whenever S comes into contact with Cd it develops integrant of co-enzymes, proteins, amino acids and defense agents e.g., GSH, amino acids, and phyto-chelatins (PCs); these defence agents are responsible for protecting plants from oxidative injury as well as damage from environmental stressors [41]. Gypsum (GYP) is another sulfur containing fertilizers. GYP application ameliorates the physical as well as the chemical attributes of soil and, in addition, denigrates the transfer of dissolved chemical compounds, particularly complex Cd, in growth media [42]. GYP is alkaline in nature, and its supplementation elevates the soil pH that accommodates the production of metal carbonates, oxides, as well as the synthesis of complexes that contribute to reduced bioavailability [43].

*Solanum nigrum* L., known as black nightshade, is an herb that is very common in wooded areas as well as disturbed habitats, and is a pioneer species that grows in polluted sites in many different countries [44]. It showed a natural ability to uptake and to accumulate large amounts of Cd in its leaves, and this is what makes it an effective Cd-phytoremediator [45,46]. In addition, *S. nigrum* is able to tolerate and concentrate high amounts of other heavy metals (Pb, Zn, Cu, Cd, Cr, and As) in aerial parts, with no apparent toxicity signs [47]. Hence, we conducted the present study to influence the effect of two different S-fertilizers (GYP, ES and *GYP+ES*) on the growth and eco-physiology of *S. nigrum* when grown in soil which was contaminated by different levels of Cd. The results of this study enhance our knowledge about (1) the enhancement of Cd phytoextraction in *S. nigrum* by using two different S-fertilizers (GYP, ES and *GYP+ES*), and (2) alterations in growth, gas exchange attributes, oxidative stress and the response of antioxidant enzymes using two different S-fertilizers (GYP, ES and *GYP+ES*) in *S. nigrum* when grown in soil which was artificially contaminated by Cd. The results from the present study give new insight that the use of gypsum (Gyp) and elemental sulfur (ES) in heavy metals in studies may be beneficial and can improve plant yield under Cd-contaminated soil.

## 2. Materials and Methods

### 2.1. Soil Sampling and Analysis

Soil was collected from the fields of University of Agriculture, Faisalabad 38000, Pakistan. Soil was sieved through 2 mm sieve to make a homogeneous mixture. Physicochemical properties of the soil included particle size, electrical conductivity (EC) and pH of the soil extract [48]. Pseudo total metals in the soil were analyzed with a standard procedure [49]. The Walkley–Black and Jackson [50] methods were used to analyzed soil organic matter, and calcium carbonate was estimated with the calcimeter method [51] and explained in Table 1.

### 2.2. Experimental Plan

This study was conducted in the Botanical Garden of Government College University, Faisalabad 38000, during April–June in 2019 under natural environmental conditions. Cadmium chloride hemi pentahydrate Cd (CdCl_2_ 5H_2_O) was purchased from Sigma-Aldrich, Inc. USA, CAS Number: 7790-78-5 to make a Cd solution for stress. The salt of CdCl_2_ was added to the soil at various concentrations (25 and 50 mg kg^−1^). We have made two distinct types of S-fertilizers (GYP and ES) with different proportions and mixed in the respective treatment (control:0, GYP 100 mg kg^−1^, ES 100 mg kg^−1^ and *GYP+ES* 50:50 mg kg^−1^) pots. Plastic pots were filled with sieved soil (5 kg pot^−1^) spiked with different Cd levels (0, 25, 50 mg kg^−1^) according to the treatment plan. The seeds of *Solanum nigrum* L. were purchased from the Ayub Agricultural Research Institute in Faisalabad, Pakistan. After sterilization (5% NaClO), seeds were soaked in deionized water for 6 h then eight seeds of *S. nigrum* were sown in plastic tray planes with quartz sand. Half strength Hoagland solution was then used for irrigation consecutively. Subsequently, after three weeks of germination, four healthy seedlings were transferred into each pot. The experiment was designed according to a completely randomized designed along with four replications of each treatment. All plants were looked up carefully, and Cd-free water and other intercultural operations were performed when needed.

### 2.3. Plant Harvesting

Plants were harvested after 70 days of transplanting the seedlings into the pots. All plants were separated into roots and shoots to measure various parameters. The diluted acid of (HCl 1.0%) was used to avoid the dirt evacuation on the surface roots after being rinsed three times with deionized water. The morphological measurements, such as shoot length, root length, shoot dry weight, root dry weight, number of leaves and leaf area, were also measured after harvesting the plants. Shoot length and root length was measured from the top of the leaf tips to the shoot, and root length was also measured. The plant samples were oven-dehydrated at 65 °C for 72 h for Cd and ions concentration determination and the total plant dry weight was also measured. Before being oven-dried, roots were immersed in 20 mM Na_2_EDTA for 15–20 min to remove Cd that had adhered to the surface of the roots. Then, the roots were washed thrice with distilled water and finally once with deionized water and dried for further analysis.

### 2.4. Determination of Photosynthetic Pigments and Gas Exchange Parameters

Leaves were collected for the determination of chlorophyll and carotenoid contents. For chlorophylls, 0.1 g of fresh leaf sample was extracted with 8 mL of 95% acetone for 24 h at 4 °C in the dark. The absorbance was measured by a spectrophotometer (UV-2550; Shimadzu, Kyoto, Japan) at 646.6, 663.6, and 450 nm. Chlorophyll content was calculated by the standard method of Arnon [52].

Gas exchange parameters were also measured during the same days. Transpiration rate, water use efficiency, photosynthetic rate and stomatal conductance were measured from four different plants in each treatment group. Measurements were conducted between 11:30 a.m. and 1:30 p.m. on days with a clear sky. Rates of leaf transpiration rate, water use efficiency, photosynthetic rate and stomatal conductance were measured with a LI-COR gas-exchange system (LI-6400; LI-COR Biosciences, Lincoln, NE, USA) with a red-blue LED light source on the leaf chamber. In the LI-COR cuvette, CO_2_ concentration was set as 380 mmol mol^−1^ and LED light intensity was set at 1000 mmol m^−2^ s^−1^, which was the average saturation intensity for photosynthesis in *S. nigrum* [53].

### 2.5. Determination of Oxidative Stress Biomarkers

Stress-induced electrolyte leakage (EL) of the uppermost stretched leaves was determined by using the methodology of Dionisio-Sese and Tobita [54]. The leaves were cut into minor slices (5 mm length) and placed in test tubes containing 8 mL distilled water. These tubes were incubated and transferred into a water bath for 2 h prior to measuring the initial electrical conductivity (EC1). The samples were autoclaved at 121 °C for 20 min and then cooled down to 25 °C before measuring the final electrical conductivity (EC2). Electrolyte leakage was calculated by the following formula; EL = (EC1/EC2) × 100

The degree of lipid peroxidation was evaluated as malondialdehyde (MDA) contents. Briefly, 0.1 g of frozen leaves were ground at 4 °C in a mortar with 25 mL of 50 mM phosphate buffer solution (pH 7.8) containing 1% polyethene pyrrole. The homogenate was centrifuged at 10,000× *g* at 4 °C for 15 min. The mixtures were heated at 100 °C for 15–30 min and then quickly cooled in an ice bath. The absorbance of the supernatant was recorded by using a spectrophotometer (xMark™ Microplate Absorbance Spectrophotometer; Bio-Rad, Hercules, CA, USA) at wavelengths of 532, 600, and 450 nm. Lipid peroxidation was expressed as 1 mol g^−1^ by using the formula: 6.45 (A532−A600)−0.56 A450. Lipid peroxidation was measured by using a method previously published by Heath and Packer [55].

To estimate the H_2_O_2_ content of plant tissues (root and leaf), 3 mL of sample extract was mixed with 1 mL of 0.1% titanium sulfate in 20% (*v/v*) H_2_SO_4_ and centrifuged at 6000× *g* for 15 min. The yellow color intensity was evaluated at 410 nm. The H_2_O_2_ level was computed by the extinction coefficient of 0.28 mmol^−1^ cm^−1^. The contents of H_2_O_2_ were measured by the method presented by Jana and Choudhuri [56].

### 2.6. Determination of Antioxidant Enzyme Activities

To evaluate enzyme activities, fresh leaves (0.5 g) were homogenized in liquid nitrogen and 5 mL of 50 mmol sodium phosphate buffer (pH 7.0), including 0.5 mmol EDTA and 0.15 mol NaCl. The homogenate was centrifuged at 12,000× *g* for 10 min at 4 °C, and the supernatant was used for measurement of superoxidase dismutase (SOD) and peroxidase (POD) activities. SOD activity was assayed in 3 mL reaction mixture containing 50 mM sodium phosphate buffer (pH 7), 56 mM nitro blue tetrazolium, 1.17 mM riboflavin, 10 mM methionine, and 100 µL enzyme extract. Finally, the sample was measured by using a spectrophotometer (xMark™ Microplate Absorbance Spectrophotometer; Bio-Rad, Hercules, CA, USA). Enzyme activity was measured by using a method by Chen and Pan [57] and expressed as U g^−1^ FW.

Peroxidase (POD) activity in the leaves was estimated by using the method of Sakharov and Ardila [58] by using guaiacol as the substrate. A reaction mixture (3 mL) containing 0.05 mL of enzyme extract, 2.75 mL of 50 mM phosphate buffer (pH 7.0), 0.1 mL of 1% H_2_O_2_, and 0.1 mL of 4% guaiacol solution was prepared. Increases in the absorbance at 470 nm because of guaiacol oxidation was recorded for 2 min. One unit of enzyme activity was defined as the amount of the enzyme.

Catalase (CAT) activity was analyzed according to Aebi [59]. The assay mixture (3.0 mL) was comprised of 100 µL enzyme extract, 100 µL H_2_O_2_ (300 mM), and 2.8 mL 50 mM phosphate buffer with 2 mM ETDA (pH 7.0). The CAT activity was measured from the decline in absorbance at 240 nm as a result of H_2_O_2_ loss (ε = 39.4 mM^−1^ cm^−1^).

Ascorbate peroxidase (APX) activity was measured according to Nakano and Asada [60]. The mixture containing 100 µL enzyme extract, 100 µL ascorbate (7.5 mM), 100 µL H_2_O_2_ (300 mM), and 2.7 mL 25 mM potassium phosphate buffer with 2 mM EDTA (pH 7.0) was used for measuring APX activity. The oxidation pattern of ascorbate was estimated from the variations in wavelength at 290 nm (ε = 2.8 mM^−1^ cm^−1^).

### 2.7. Cd Determination in Plants

The grounded samples were digested with pure HNO_3_ at 190 °C for 45 min (10 min pre-heating, 15 min heating, 20 min cooling) in a microwave oven (Mars 6, CEM Corporation, Matthews, NC, USA) with the settings described in detail by Jezek et al. [61]. Samples were diluted with 2% HNO_3_ and determined by an Agilent 240FS-AA atomic absorption spectrophotometer (AAS).

### 2.8. Statistical Analysis

The statistical analysis of the data was performed with analysis of variance (ANOVA) by using a statistical program Co-Stat version 6.2, Cohorts Software, 2003, Monterey, CA, USA. All the data obtained were tested by two-way analysis of variance (ANOVA). Thus, the differences between treatments were determined by using ANOVA, and the least significant difference test (*p* < 0.05) was used for multiple comparisons between treatment means. Logarithmic or inverse transformations were performed for data normalization, where necessary, prior to analysis. Pearson’s correlation analysis was performed to quantify relationships between various analyzed variables. The Pearson correlation coefficients and principal component analysis (PCA) between the measured variables of *S. nigrum* were also calculated with Rstudio software.

## 3. Result

### 3.1. Effect of S-Fertilizer on Biomass and Growth of the Plants under Cd Contaminated Soil

**Figure 1:**
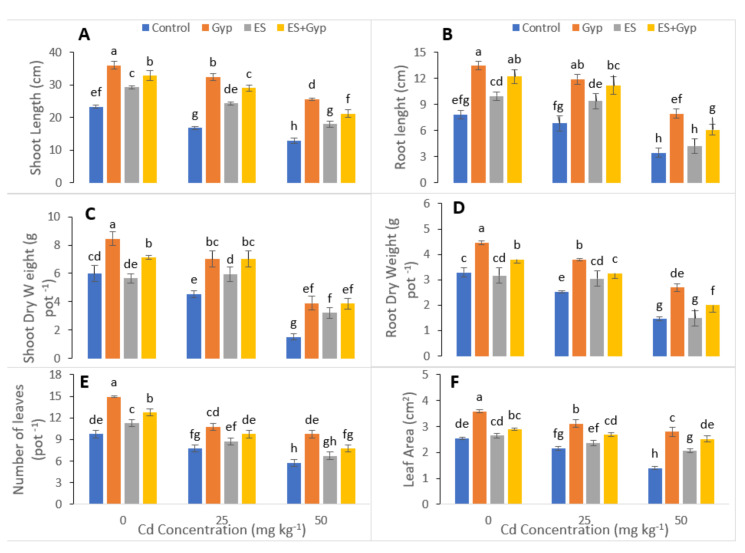
Effect of different concentrations of Cd (25, 50 mg kg^−1^) in the soil on growth related attributes i.e., shoot length (**A**), root length (**B**), shoot dry weight (**C**), root dry weight (**D**), number of leaves (**E**) and leaf area (**F**) of *S. nigrum* grown under the S-Fertilizers, alone GYP, ES and in combination *GYP+ES*. S-Fertilizer showed significant increase in growth parameters compared to control. Bars sharing different letter(s) for each parameter are significantly different from each other according to Duncan’s multiple rang test (*p* ˂ 0.05). All the data represented are the average of four replications.

In the present study, we have also measured shoot length, root length, shoot and root dry weight, number of leaves and leaf area in *S. nigrum* under the S-Fertilizers, alone GYP, ES and in combination *GYP+ES* which is presented in Figure 1. Our results revealed that the increasing levels of Cd (25 and 50 mg kg^−1^) in the soil significantly (*p* < 0.05) decreased shoot length, root length, shoot and root dry weight, number of leaves and leaf area, compared to the plants which were grown in the controlled treatment i.e., without the addition of Cd in the soil. However, the results also indicated that the S-fertilizers, alone with GYP, and ES and in combination with *GYP+ES* significantly (*p* < 0.05) increased shoot length, root length, shoot and root dry weight, number of leaves and leaf area of *S. nigrum* compared to the plants which were not treated with the application of *GYP+ES*. However, the plants treated with GYP showed maximum growth and biomass compared to ES and control at different Cd (25, 50 mg kg^−1)^ concentrations in the soil. In addition, the maximum root and shoot lengths were increased in the plants which were grown in the application of gypsum (100 mg kg^−1^) compared to ES (100 mg kg^−1^) and control. Similar results were obtained by studying root, shoot dry biomass, number of leaves and leaf area with the application of gypsum (100 mg kg^−1^), and this increased the shoot (57, 54, 154%), root (57, 68, 106%), number of leaves (66, 59, 105%) and leaf area (48, 57, 100%) of the dry weight as compared to ES and control, respectively.

### 3.2. Effect of S-Fertilizer on Photosynthetic Pigments and Gas Exchange Characteristics of the Plants under Cd Contaminated Soil

**Figure 2:**
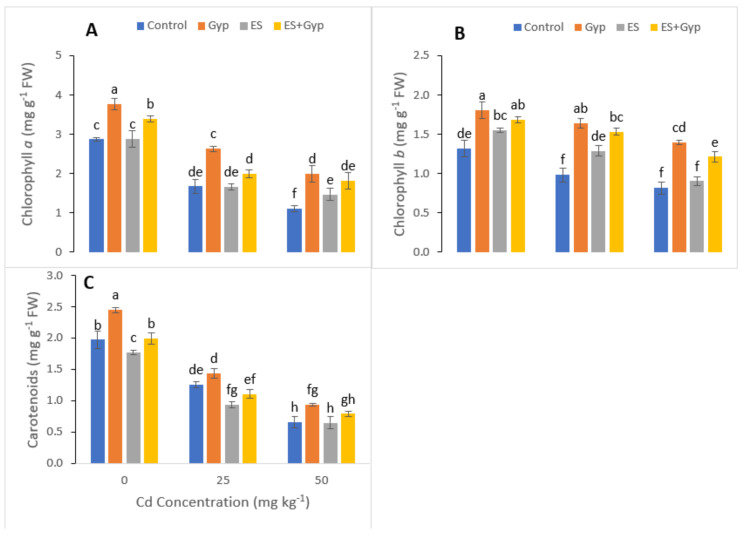
Effect of different concentrations of Cd (25, 50 mg kg^−1^) in the soil on photosynthetic pigments i.e., chlorophyll a (**A**), chlorophyll b (**B**) and carotenoid (**C**) of *S. nigrum* grown under the S-fertilizers, separate from GYP, ES and in combination with *GYP+ES*. S-fertilizer showed significant increase in growth parameters compared to control. Bars sharing different letter(s) for each parameter are significantly different from each other according to Duncan’s multiple rang test (*p* ˂ 0.05). All the data represented are the average of four replications.

The individual and combined application of GYP and ES increased the chlorophyll contents and gas exchange characteristics in leaves of *S. nigrum* as shown in Figure 2. Results showed that Cd toxicity (25 and 50 mg kg^−1^) decreased chlorophyll a, chlorophyll b and carotenoid content in *S. nigrum*. However, chlorophyll a, chlorophyll b and carotenoid content were increased under the application of GYP (Figure 2)., Maximum concentration was observed in the plants GYP and in the combination of *GYP+ES* application as compared to ES application alone and control at different Cd levels. Results showed that the individual application of GYP increased the concentration of chlorophyll a, chlorophyll b and carotenoid content by 67, 99, 105% compared to the control, respectively.

**Figure 3:**
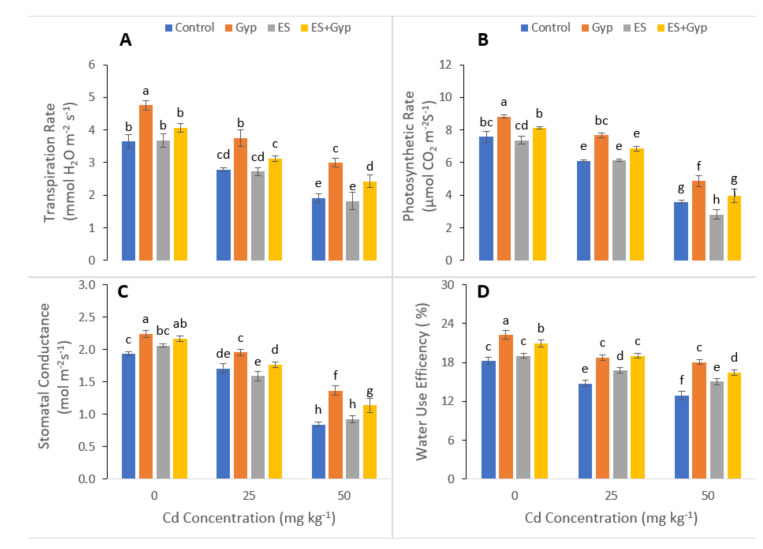
Effect of different concentrations of Cd (25, 50 mg kg^−1^) in the soil on photosynthetic pigments i.e., transpiration rate (**A**), photosynthetic rate (**B**), stomatal conductance (**C**), and water use efficiency (**D**) of S. nigrum grown under the S-fertilizers, separate from GYP, ES and in combination with *GYP+ES*. S-fertilizer showed significant increase in growth parameters compared to control. Bars sharing different letter(s) for each parameter are significantly different from each other according to Duncan’s multiple rang test (*p* ˂ 0.05). All the data represented are the average of four replications.

In the present study, various gas exchange characteristics such as transpiration rate, photosynthetic rate, stomatal conductance and water use efficiency were also measured in *S. nigrum* grown under the toxic level of Cd (25 and 50 mg kg^−1^) in the soil with or without the application of GYP, ES (Figure 3). From the given results, it can be shown that the increasing levels of Cd in the soil significantly (*p* < 0.05) decreased transpiration rate, photosynthetic rate, stomatal conductance and water use efficiency in *S. nigrum*. However, these attributes can be increased by the application of GYP and ES in individually or in combination (Figure 3). Results showed that transpiration rate, photosynthetic rate, stomatal conductance and water use efficiency were increased by 55, 20, 32 and 18%, respectively, under the application of GYP, while they also increased by 23, 8, 13, and 14%, respectively, under the application of ES, and further increased by 41, 14, 33 and 24%, respectively, under the combined application of ES and GYP.

### 3.3. Effect of S-Fertilizer on Oxidative Stress Indicators and Response of Antioxidative Enzymes of the Plants under Cd Contaminated Soil

**Figure 4:**
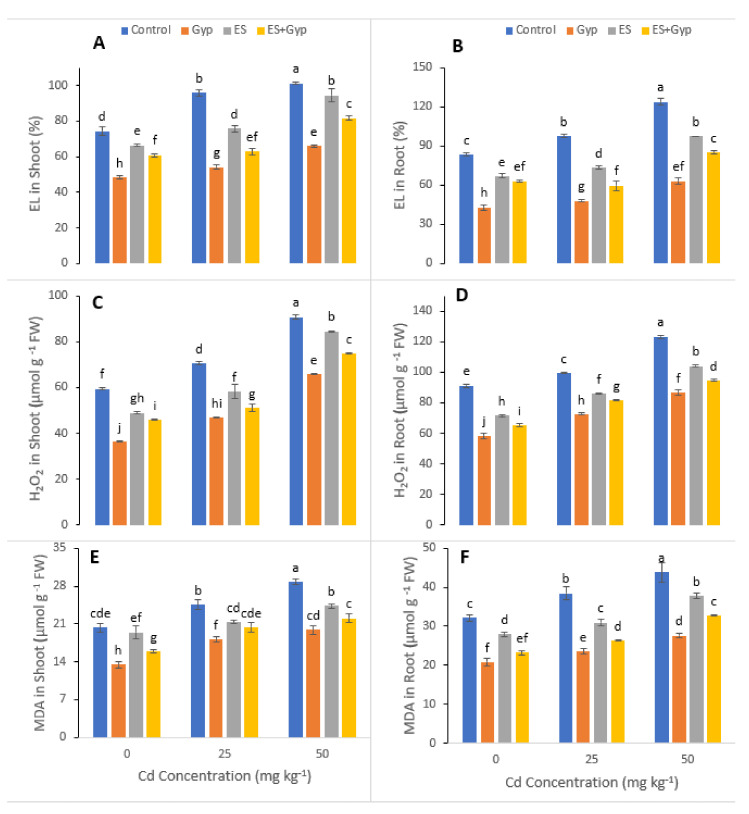
Effect of different concentrations of Cd (25, 50 mg kg^−1^) in the soil on oxidative stress biomarkers i.e., electrolyte leakage in the shoots (**A**), electrolyte leakage in the roots (**B**), hydrogen peroxide content in the shoots (**C**), hydrogen peroxide content in the roots (**D**), malondialdehyde contents in the shoots (**E**) and malondialdehyde contents in the roots (**F**) of *S. nigrum* grown under the S-Fertilizers, alone with GYP, and ES and in combination *GYP+ES*. S-fertilizer showed significant increase in growth parameters compared to control. Bars sharing different letter(s) for each parameter are significantly different from each other according to Duncan’s multiple range test (*p* < 0.05). All the data represented are the average of four replications.

In the present study, oxidative stress biomarkers such as malondialdehyde (MDA), hydrogen peroxide (H_2_O_2_), and electrolyte leakage (EL) were also measured in *S. nigrum* grown under the S-fertilizers, separate from GYP and ES and in combination with *GYP+ES* in the soil which was artificially contaminated with Cd at various levels (25, 50 mg kg^−1^). The data regarding various oxidative stress biomarkers is presented in Figure 4. According to the results, Cd toxicity significantly (*p* < 0.05) increased MDA, H_2_O_2_, and EL, while the maximum increase of MDA, H_2_O_2_, and EL was observed in the plants which were grown under the treatment of 50 mg kg^−1^ without the application of GYP and ES. Results also showed that the application of GYP and ES individually or in combination significantly (*p* < 0.05) decreased the concentration of oxidative stress biomarkers (Figure 4). The application of GYP, ES and the combined application of GYP and ES decreased the MDA contents by 25, 40, 30% in leaves and 13, 43, and 22% in roots, respectively. Furthermore, the application of GYP, ES and the combined application of GYP decreased H_2_O_2_ contents by 53, 23, and 42% and decreased these contents in roots by 48, 35, and 39%, respectively. Similarly, the application of GYP, ES and the combined application of GYP decreased EL contents by 14, 36, and 18% in leaves and 20, 36, 24% in roots, respectively.

**Figure 5:**
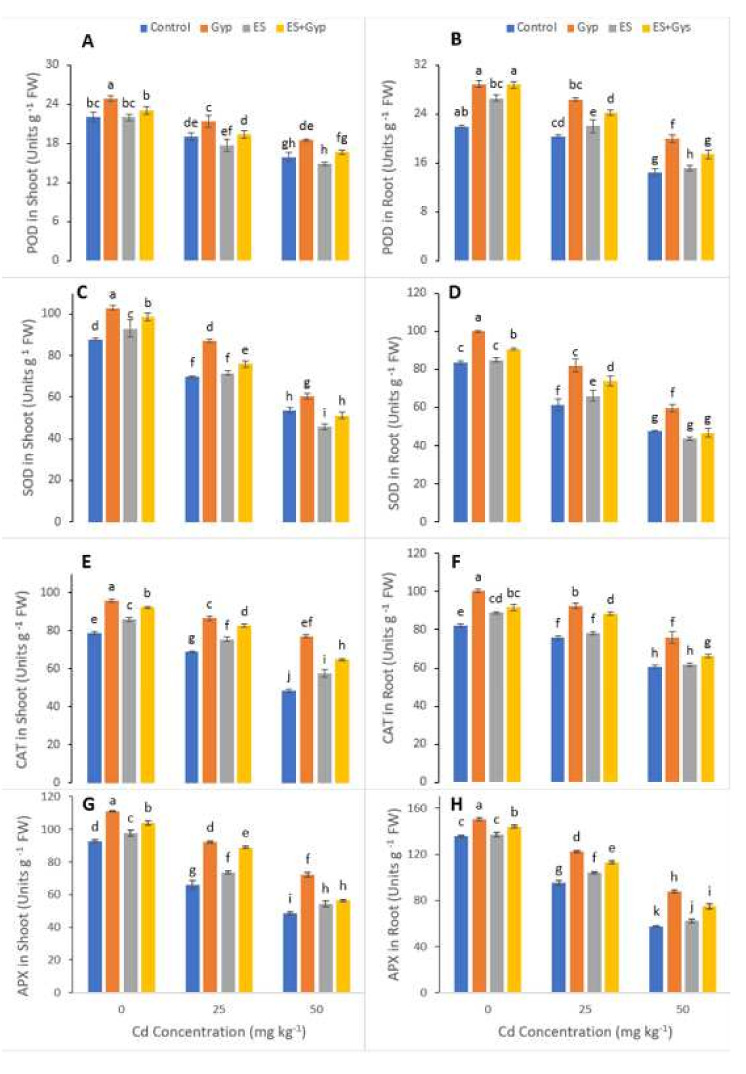
Effect of different concentrations of Cd (25, 50 mg kg^−1^) in the soil on antioxidant capacity i.e., peroxide activity in the shoots (**A**), peroxide activity in the roots (**B**), superoxidase activity in the shoots (**C**), superoxidase activity in the roots (**D**), catalase activity in the shoots (**E**) catalase activity in the roots (**F**), ascorbate peroxidase activity in the shoots (**G**) and ascorbate peroxidase in the roots (**H**) of *S. nigrum* grown under the S-Fertilizers, alone GYP, ES and in combination *GYP+ES*. S-Fertilizer showed significant increase in growth parameters compared to control. Bars sharing different letter(s) for each parameter are significantly different from each other according to Duncan’s multiple range test (*p* < 0.05). All the data represented are the average of four replications.

The various activities of antioxidants such as superoxide dismutase (SOD), peroxidase (POD) and catalase (CAT) and ascorbate peroxidase (APX) were also measured in the present study and are shown in Figure 5. Increasing levels of Cd (25, 50 mg kg^−1^) in the soil significantly (*p* < 0.05) decreased the activities of POD, SOD, CAT and APX comapped to those plants which were grown in the control treatment. Results also showed that the application of GYP and ES individually or combined significantly (*p* < 0.05) increased the activities of various antioxidant compounds compared to the control treatment. However, the maximum increase was observed in the plants which were grown in the individual application of GYP compared to the all other treatments of the experiment (Figure 5). The plants grown in the application of GYP increased the activities of POD, SOD, CAT and APX by 31, 52, 52, and 47%, respectively, in the shoots, while also increasing by 35, 78, 80 and 60%, respectively, in the roots.

### 3.4. Effect of S-Fertilizer on Cd Accumulation of the Plants under Cd Contaminated Soil

**Figure 6:**
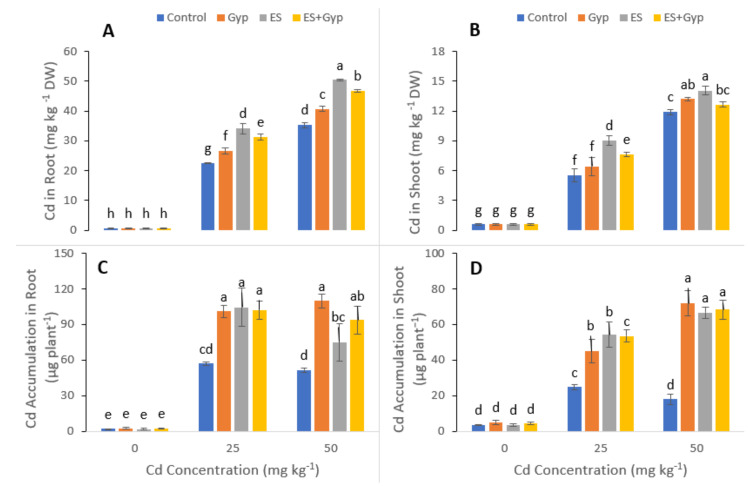
Effect of different concentrations of Cd (25, 50 mg kg^−1^) in the soil on Cd uptake and accumulation i.e., Cd concentration in the shoots (**A**), Cd concentration in the roots (**B**), Cd accumulation in the shoots (**C**) and Cd accumulation in the roots (**D**) of *S. nigrum* grown under the S-fertilizers, alone with GYP, and ES and in combination with *GYP+ES*. S-fertilizer showed a significant increase in growth parameters compared to control. Bars sharing different letter(s) for each parameter are significantly different from each other according to Duncan’s multiple range test (*p* < 0.05). All the data represented are the average of four replications.

The Cd uptake and accumulation in various parts of the plants (roots and shoots) were also measured in *S. nigrum*, when grown under the different levels of Cd in the soil (25, 50 mg kg^−1^) with or without the application of S-Fertilizers, alone GYP, ES and in combination with *GYP+ES*. The data regarding Cd uptake and Cd accumulation is presented in Figure 6. It was observed that the increasing levels of Cd in the soil significantly (*p* < 0.05) increased the Cd concentration in the different parts of the plants without the application of S-fertilization. However, our results also illustrated that the application of GYP and ES further increased the concentration of Cd in the roots and shoots of the plants (Figure 6). The maximum concentration of Cd in the roots and shoots was observed in the plants which were grown under the application of ES compared to GYP alone and in combination with *GYP+ES* in roots and shoots.

### 3.5. Correlation between Various Growth and Physiological Parameters with Cd Uptake and Accumulation

**Figure 7:**
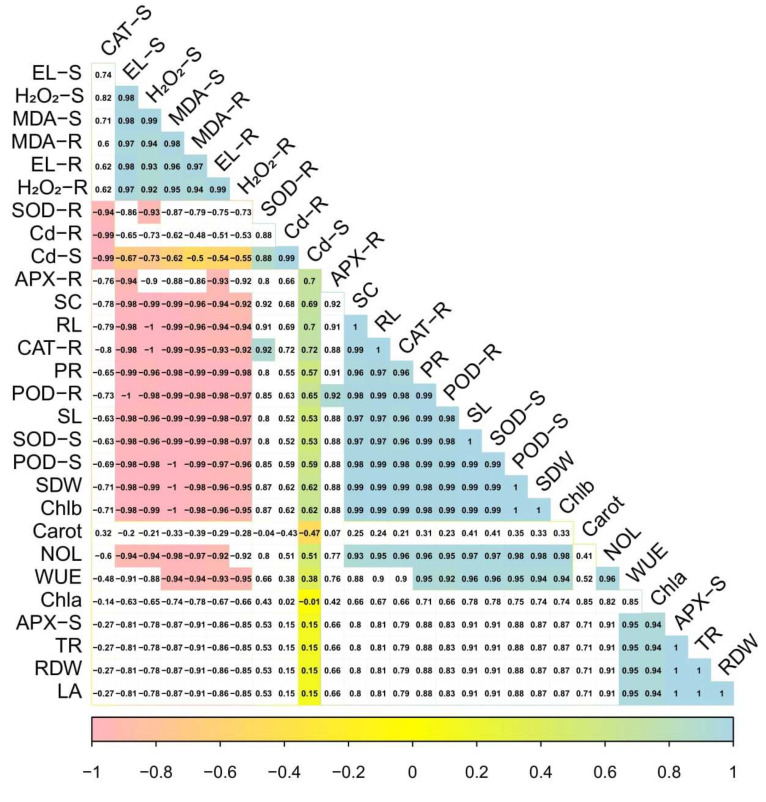
Correlation between growth and physiological parameters of *S. nigrum* grown under Cd contaminated soil with the application of S-Fertilizers. Different abbreviations used in the figure are as follows: Cd-R (Cd concentration in the roots) Cd-S (Cd concentration in the shoots), SL (shoot length), RL (root length), SDW (shoot dry weight), RDW (root dry weight), NOL (number of leaves), LA (leaf area), Chla (chlorophyll a), Chlb (chlorophyll b), Carrot (carotenoid contents), TR (transpiration rate), WUE (water use efficiency), PR (photosynthetic rate), SC (stomatal conductance), POD-S (peroxide activity in the shoots), POD-R (peroxide activity in the roots), SOD-S (superoxidase activity in the shoots), SOD-R (superoxidase activity in the roots), CAT-S (catalase activity in the shoots), CAT-R (catalase activity in the roots), APX-S (ascorbate peroxidase activity in the shoots), APX-R (ascorbate peroxidase in the roots) EL-S (electrolyte leakage in the shoots), EL-R (electrolyte leakage in the roots), H_2_O_2_-S (hydrogen peroxide content in the shoots), H_2_O_2_-R (hydrogen peroxide content in the roots), MDA-S (malondialdehyde contents in the shoots) and MDA-R (malondialdehyde contents in the roots).

A Pearson’s correlation also quantified a relationship with growth and physiological parameters of *S. nigrum* grown under Cd contaminated soil with the application of S-fertilizers (Figure 7). Cd concentration in the roots was positively correlated with Cd concentration in the shoots, shoot length, root length, shoot dry weight, root dry weight, number of leaves, leaf area, chlorophyll a, chlorophyll b, carotenoid contents, transpiration rate, water use efficiency, photosynthetic rate, stomatal conductance, peroxide activity in the shoots, peroxide activity in the roots, superoxidase activity in the shoots, superoxidase activity in the roots, catalase activity in the shoots, catalase activity in the roots, ascorbate peroxidase activity in the shoots, and ascorbate peroxidase in the roots, while negatively correlated with electrolyte leakage in the shoots, electrolyte leakage in the roots, hydrogen peroxide content in the shoots, hydrogen peroxide content in the roots, malondialdehyde contents in the shoots and malondialdehyde contents in the roots. Similarly, Cd concentration in the shoots was positively correlated with Cd concentration in the roots, shoot length, root length, shoot dry weight, root dry weight, number of leaves, leaf area, chlorophyll a, chlorophyll b, carotenoid contents, transpiration rate, water use efficiency, photosynthetic rate, stomatal conductance, peroxide activity in the shoots, peroxide activity in the roots, superoxidase activity in the shoots, superoxidase activity in the roots, catalase activity in the shoots, catalase activity in the roots, ascorbate peroxidase activity in the shoots, and ascorbate peroxidase in the roots while negatively correlated with electrolyte leakage in the shoots, electrolyte leakage in the roots, hydrogen peroxide content in the shoots, hydrogen peroxide content in the roots, malondialdehyde contents in the shoots and malondialdehyde contents in the roots. This relationship showed a close connection between growth and Cd uptake in *S. nigrum*.

### 3.6. Principal Component Analysis

**Figure 8:**
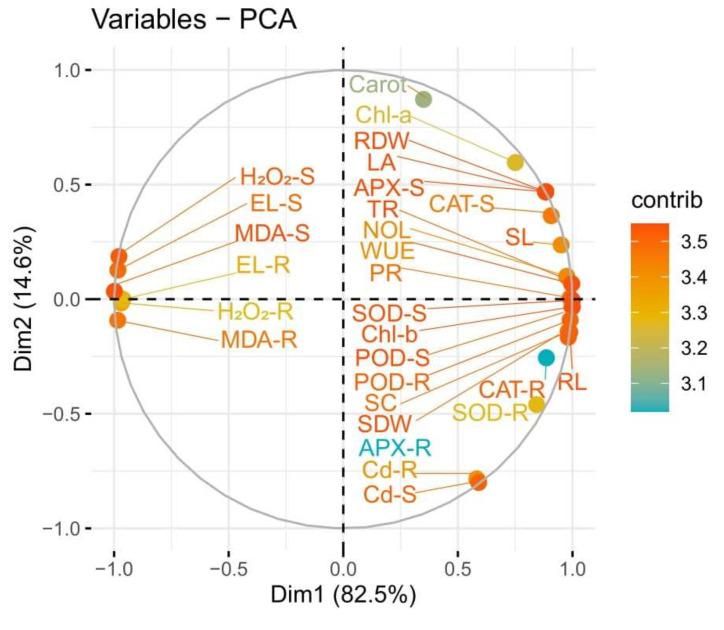
Loading plots of principal component analysis showed a relationship between growth and physiological parameters of *S. nigrum* grown under Cd contaminated soil with the application of S-Fertilizers. Different abbreviations used in the figure are as follow: Cd-R (Cd concentration in the roots) Cd-S (Cd concentration in the shoots), SL (shoot length), RL (root length), SDW (shoot dry weight), RDW (root dry weight), NOL (number of leaves), LA (leaf area), Chla (chlorophyll a), Chlb (chlorophyll b), Carrot (carotenoid contents), TR (transpiration rate), WUE (water use efficiency), PR (photosynthetic rate), SC (stomatal conductance), POD-S (peroxide activity in the shoots), POD-R (peroxide activity in the roots), SOD-S (superoxidase activity in the shoots), SOD-R (superoxidase activity in the roots), CAT-S (catalase activity in the shoots), CAT-R (catalase activity in the roots), APX-S (ascorbate peroxidase activity in the shoots), APX-R (ascorbate peroxidase in the roots) EL-S (electrolyte leakage in the shoots), EL-R (electrolyte leakage in the roots), H_2_O_2_-S (hydrogen peroxide content in the shoots), H_2_O_2_-R (hydrogen peroxide content in the roots), MDA-S (malondialdehyde contents in the shoots) and MDA-R (malondialdehyde contents in the roots).

The loading plots of principal component analysis (PCA) were also constructed to illustrate a relationship between the growth and physiological parameters of *S. nigrum* grown under Cd contaminated soil with the application of S-fertilizers (Figure 8). Of all the main components, the first two components, Dim1 and Dim2, comprise more than 97% of the whole database and make up the largest portion of all components. Among this, Dim1 contributes 82.5%, and Dim2 contributes 14.6% of the whole dataset. The first group of variables with which PC1 is positively correlated includes: Cd concentration in the roots, Cd concentration in the shoots, shoot length, root length, shoot dry weight, root dry weight, the number of leaves, leaf area, chlorophyll a, chlorophyll b, carotenoid contents, transpiration rate, water use efficiency, photosynthetic rate, stomatal conductance, peroxide activity in the shoots, peroxide activity in the roots, superoxidase activity in the shoots, superoxidase activity in the roots, catalase activity in the shoots, catalase activity in the roots, ascorbate peroxidase activity in the shoots, and ascorbate peroxidase in the roots. A significant negative correlation of PC1 variables was found with the variables aligned with electrolyte leakage in the shoots, electrolyte leakage in the roots, hydrogen peroxide content in the shoots, hydrogen peroxide content in the roots, malondialdehyde contents in the shoots and malondialdehyde contents in the roots.

## 4. Discussion

Inorganic amendments were changed in this study with the same concentration in a previous experiment [62] with the same plant *S. nigrum.* The results showed that GYP and ES application at different levels increased the shoot length, root length, shoot and root dry weight, the number of leaves, as well as the leaf area compared to the plants which were not treated with GYP and ES (Figure 1). A similar result was shown by Qayyum et al. [42] that GYP and MAP application significantly (*p* < 0.05) increased grain yield in wheat compared to the control with the increase of the dose-additive. However, Cd is non-vital for plant development, but is easily taken up by plant roots and shoots, which makes it a metal of utmost concern regarding its accumulation in the food chain [24,32]. Photosynthesis, respiration, cell division, water relations, opening and closing of stomata, nitrogen metabolism, and mineral nutrition are the main metabolic processes within the plants, which are negatively affected by Cd stress [31,62]. Cd reduces the photosynthetic capacity of plants by devastating the enzymes of the Calvin cycle and the carbohydrate metabolism and also modulates the antioxidant machinery of the plants. All these physiological changes result in decreased plant yield [15,23,63]. A number of studies have been documented, and the results revealed that Cd toxicity seized the plant growth as well as the developmental process, and subsequently destroyed and damaged the metabolic and physiological function of plants, for instance, respiration, photosynthesis, chlorophyll content, stomatal conductance, nutrient imbalance as well as nitrogen metabolism [12,15,18,63]. It is well known that Cd toxicity in crops depends on the bioavailability of Cd in soils and the concentration of elements, which can compete with Cd during plant uptake [20,62,64]. In the present study we have noticed that the gypsum that immobilized the Cd uptake improved *S. nigrum* growth parameters, while the elemental sulfur application improved Cd uptake in this study (Figure 1 and Figure 6). Khan et al. [65] reported that sulfur has the capacity to increase metal mobility in wheat. Qayyum et al. [42] had reported that sulfur application significantly increased plant growth and biomass in wheat plants under a stressed environment.

The gas exchange constitutes an important parameter for evaluation of the photosynthetic activity, which is crucial to determining the adaptation and stability of plants subjected to different environmental adversities, as the variation in photosynthetic rates implies alterations in growth as well as productivity [66,67,68]. Furthermore, gas exchange characteristics are considered as effective physiological indicators, which could be used to assess the intensity of stress on plants grown under metal toxicity [69,70]. Excessive Cd concentrations affected the net photosynthesis due to two important factors; (i) stomatal factors and (ii) non-stomatal factors. Ascorbic acid mediated the closure of the stomata under the excess concentration of Cd causes a reduction in stomatal numbers under the influence of stomatal factors [62,71]. Non-stomatal considerations, however, include limitations of net photosynthesis, probably due to the reduction of different enzymes involved in chlorophyll synthesis, as well as Calvin cycle inhibition, and also phosphoenolpyruvate carboxylase in C4 plants [6,72,73,74]. In this study, it has been observed that the highest Cd toxicity decreased chlorophyll a, b, and total chlorophyll in *S. nigrum* leaves, compared to the control (Figure 2). The application of Sulphur increased photosynthetic efficiency and gas exchange attributes, which was observed at an extent in the leaves of *S. nigrum* compared to the control (Figure 2). Similar findings were observed by Hasan et al. [75] when they studied tomato under stress conditions.

**Figure 9:**
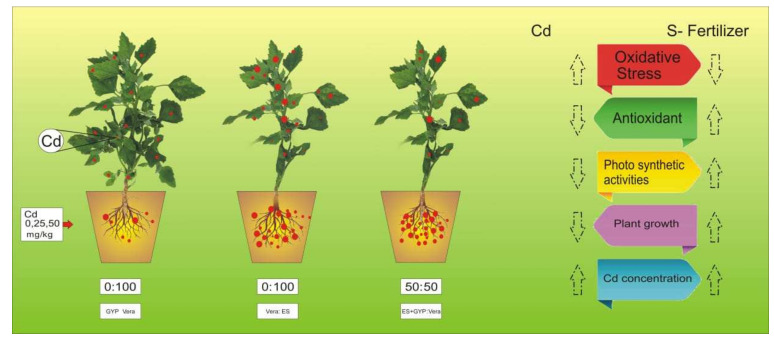
Schematic presentation of Cd toxicity in *S. nigrum* under the application of S-fertilizers (Gyp, ES and ES+Gyp).

Heavy metals are considered a primary source of injury to the cell membrane, and this is frequently attributed to lipid peroxidation. As a result of metal accumulation, a large number of active free oxygen radicals are formed, which may be the main cause of cell membrane lipid peroxidation, and this also harms the functioning and structure of the cell membrane [76,77,78]. Excessive reactive oxygen species (ROS) production causes oxidative stress, as reported for many crops under heavy metals treatment, and is likely to be commenced by molecular oxygen excitation (O_2_), to generate singlet oxygen, or by electron transfer to O2 and the genesis of free radicals, i.e., O^2−^ and OH^−^ [79,80,81]. Plant response to oxidative stress also depends upon plant species and cultivars, and this ROS production in plants is removed by a variety of antioxidant enzymes such as SOD, POD, CAT, and APX [82,83]. However, the external application with S-Fertilizer significantly (*p* < 0.05) decreased O^2−^, H_2_O_2_, and MDA (Figure 4), and also increased the activities of antioxidant enzymes such as SOD, POD, CAT, and APX (Figure 5) in the roots and shoots of the plants. Plants developed complex defense systems to cope with ROS at the cell level. APX, SOD, POD, and CAT enzymes are known as defenders of antioxidant enzyme systems which protect plants from ROS species. It was observed that higher Cd concentration increased the activities of SOD, CAT, and POD in wheat and rice [18,84]. The present study showed that S-fertilizer significantly (*p* < 0.05) increased the antioxidant enzymes such as SOD, POD, CAT, and APX in shoots and roots of *S. nigrum* (Figure 5). The sulfur supplementation has the potential to cope with the plant growth in addition to the buildup of sulfate into plant tissues, and could improve the tolerance of plants compared to stresses like Cd toxicity [42]. The application of GYP improves growth parameters while the ES enhanced Cd uptake would be a promising approach to have maximum yield and to clean the environment, as shown in Figure 6. Overall, the result showed that different proportions of S-fertilizer significantly increased the growth, composition and phytoextraction of Cd when the plant was grown in Cd-contaminated soil. The schematic presentation of Cd toxicity and also the effect of S-fertilizer is shown in Figure 9.

## 5. Conclusions

In the present study, we concluded that the optimum application of S-fertilizers (Gyp, ES and ES+Gyp) have the potential to ameliorate the Cd toxicity, using *S. nigrum,* when grown in the Cd-contaminated soil. The toxic level of Cd significantly affected plant growth and biomass, photosynthetic pigments, gaseous exchange traits, and antioxidative machinery in *S. nigrum*. Furthermore, Cd toxicity increased the oxidative stress indicators and Cd contents in plant organs. GYP application increased plant growth and composition as compared to ES and their combined application, *GYP+ES*. On the other hand, ES increased Cd uptake by *S. nigrum* as compared to GYP and *GYP+ES*. However, ES (100 mg kg^−1^) might be the productive approach to decontaminate the heavy metals polluted soil through *S. nigrum*. In addition, GYP (100 mg kg^−1^) might be a productive approach to increasing the yield and biomass of *S. nigrum* in Cd-contaminated soil. Thus, both GYP and ES are the best supplementations of S-fertilizers and would be effective in phytoremediation of heavy metals by *S. nigrum*.

## Figures and Tables

**Table 1:** Physico-chemical analysis of soil used for this experiment.

Soil	Units
Textural Class	Sandy Clay Loam
Sand	63.7%
Silt	14.4%
Clay	21.9%
pH	7.71
EC	1.93 dS m^−1^
HCO_3_^−1^	3.1 mmol L^−1^
Total nitrogen	0.06%
Available P	2.7 mg kg^−1^
K^+^	0.08 mmol L^−1^
Cl^−1^	5 mmol L^−1^
Ca^+2^ + Mg^+2^	14.34 mmol L^−1^
Available Cd	0.03 mg kg^−1^

## Data Availability

Data is contained within the article.
